# Correction: Shinohara et al. Relationship Between Diaphragm Function and Sarcopenia Assessed by Ultrasound: A Cross-Sectional Study. *Diagnostics* 2025, *15*, 90

**DOI:** 10.3390/diagnostics15121522

**Published:** 2025-06-16

**Authors:** Takahiro Shinohara, Toru Yamada, Shuji Ouchi, Suguru Mabuchi, Ryoichi Hanazawa, Kazuharu Nakagawa, Kanako Yoshimi, Tatsuya Mayama, Ayane Horike, Kenji Toyoshima, Yoshiaki Tamura, Atsushi Araki, Haruka Tohara, Akihiro Hirakawa, Takuma Kimura, Takeshi Ishida, Masayoshi Hashimoto

**Affiliations:** 1 Department of General Medicine, Graduate School of Medical and Dental Sciences, Institute of Science Tokyo, Tokyo 113-8510, Japan; tshifmed@tmd.ac.jp (T.S.); ouchi.vasc@tmd.ac.jp (S.O.); mabuchi.vasc@tmd.ac.jp (S.M.); mikejpn007@gmail.com (M.H.); 2 Department of Clinical Biostatistics, Graduate School of Medical and Dental Sciences, Institute of Science Tokyo, Tokyo 113-8510, Japan; r-hanazawa.crc@tmd.ac.jp (R.H.); a-hirakawa.crc@tmd.ac.jp (A.H.); 3 Department of Dysphagia Rehabilitation, Department of Gerontology and Gerodontology, Graduate School of Medical and Dental Sciences, Institute of Science Tokyo, Tokyo 113-8510, Japan; k.nakagawa.swal@tmd.ac.jp (K.N.); k.yoshimi.gerd@tmd.ac.jp (K.Y.); gambatteiki@gmail.com (T.M.); ayanehorike55@gmail.com (A.H.); harukatohara@hotmail.com (H.T.); 4 Department of Diabetes, Metabolism, and Endocrinology, Tokyo Metropolitan Institute for Geriatrics and Gerontology, Tokyo 173-0015, Japan; kenji_toyoshima@tmghig.jp (K.T.); tamurayo@tmghig.jp (Y.T.); aaraki@tmghig.jp (A.A.); 5 Department of R&D Innovation for Home Care Medicine, Graduate School of Medical and Dental Sciences, Institute of Science Tokyo, Tokyo 152-8550, Japan; kimura.takuma@tmd.ac.jp; 6 Department of Community Medicine (Ibaraki), Graduate School of Medical and Dental Sciences, Institute of Science Tokyo, Tokyo 152-8550, Japan; ishida.takeshi@tmd.ac.jp

Upon reviewing our original publication [1], we noticed some minor errors in the text, so we would like to request a few corrections. These are localized errors and do not affect our analytical results or conclusions.

## 1. Text Correction in Abstract

There was an error in the Abstract of the original publication. We had mistakenly reported values calculated from a total of 148 registered cases. Although 148 cases were registered, sarcopenia assessments could not be conducted in 10 of them, resulting in a final analysis of 138 cases. We have also corrected the reported age and gender proportions to reflect calculations based on the 138 analyzable cases. We have confirmed that this correction does not affect any other results.

A correction has been made to the results part of the Abstract:

**Abstract: Background/Objectives**: The diaphragm is important for respiration, but the effects of age-related muscle loss and sarcopenia on diaphragm function are unclear. We evaluated the associations of sarcopenia and skeletal muscle mass (SMM) with diaphragm function. **Methods**: This study was conducted at three Japanese hospitals from May 2023 to September 2024. The participants underwent bioelectrical impedance for SMM assessment, as well as pulmonary function tests. Diaphragm ultrasound was used to measure the thickness at functional residual capacity (FRC), thickening fraction (TF), and diaphragm excursion (DE) during deep breathing (DB), and their associations with sarcopenia and low skeletal muscle index (SMI) were analyzed. **Results**: Overall, 138 patients (mean age 78.0 years; sarcopenia, *n* = 35; non-sarcopenia, *n* = 103) were included. No statistically significant differences in thickness (FRC), TF and DE were observed between the sarcopenia group and the non-sarcopenia group. The low SMI group had significantly lower thickness (difference −0.22, 95% CI; −0.41, −0.29) and DE (difference −9.2, 95%CI; −14.0, −4.49) than the normal SMI group. Multivariable linear regression analyses adjusted for age, sex, and stature revealed no association between thickness (FRC) and sarcopenia (*p* = 0.98), but thickness (FRC) was negatively associated with low SMI (*p* = 0.034). DE during DB was negatively associated with sarcopenia (*p* = 0.024) and low SMI (*p* = 0.001). TF showed no associations. **Conclusions**: DE during DB was reduced in patients with sarcopenia and low SMI, and thickness (FRC) was reduced in those with low SMI without sarcopenia.

## 2. Text Correction in Results

There was an error in the original publication. The reported age and female proportion contain errors.

A correction has been made to the Results, paragraph 1:

The mean participant age was 78.0 (standard deviation of 6.9) years, and 52.2% of the participants were female. There were no significant differences in sex, Brinkman Index, Charlson Comorbidity Index, Barthel Index, stature, forced expiratory volume in 1 s/forced vital capacity ratio, and peak expiratory flow rate between the sarcopenia group and the non-sarcopenia group. The sarcopenia group was older and had a lower Mini-Mental State Examination score, lower body mass, lower grip strength, and slower walking speed than the non-sarcopenia group. Additionally, the percent vital capacity, as an indicator of respiratory function, was lower in the sarcopenia group (Table 1).

## 3. Error in Tables 1–5

In the original publication, there was a mistake in “Table 1. Participants’ characteristics” as published. There was one “No answer” response regarding smoking history in the non-sarcopenia group, but it was not reflected in the table. The corrected Table 1 appears below.

In the original publication, there was a mistake in “Table 2. Comparison of diaphragm thickness, thickening fraction, and diaphragm excursion between non-sarcopenia and sarcopenia groups.” as published. The values had not been correctly rounded, so we corrected them (e.g., from 0.021 to 0.02). The corrected Table 2 appears below.

In the original publication, there was a mistake in “Table 3(b). Multiple regression analysis for each right hemidiaphragm measurement with adjustment for independent variables.” as published. The values had not been correctly rounded, and we have now corrected the rounding error (e.g., from 0.86 to 0.85). The corrected Table 3(b) appears below.

In the original publication, there was a mistake in “Table 4. Comparison of diaphragm thickness, thickening fraction, and diaphragm excursion between normal SMI and low SMI groups.” as published. Incorrect values were mistakenly input into the “Total” column during the data transfer process. We have corrected these errors and confirmed that all other values in the table are accurate. The corrected Table 4 appears below.

In the original publication, there was a mistake in “Table 5(b). Multiple regression analysis for each right hemidiaphragm measurement with adjustment for independent variables” as published. Incorrect coefficient and *p*-value results were recorded for Age and FRC during data transfer, and these have now been corrected. Additionally, the coefficients for the thickening fraction and low SMI were not properly rounded, so we revised them (e.g., from 17.1 to 17.2). Furthermore, during communication with the Editorial Office, the “Stature” item in Table 5(b) was mistakenly deleted; this has also been corrected. The corrected Table 5(b) appears below. 

The authors state that their scientific conclusions are unaffected. This correction was approved by the Academic Editor. The original publication has also been updated.

## Figures and Tables

**Table 1:** Participants’ characteristics.

	Non-Sarcopenia (*n* = 103)	Sarcopenia (*n* = 35)	*p* Value
Age (years), mean (SD)	76.6 (6.81)	81.9 (5.36)	<0.001
Female, *n* (%)	56 (54.4)	16 (45.7)	0.49
Never smoker, *n* (%)	59 (57.2)	22 (62.9)	
Current smoker, *n* (%)	7 (6.8)	2 (5.7)	
Past smoker, *n* (%)	36 (35.0)	11 (31.4)	
No answer, *n* (%)	1 (1.0)	0 (0.0)	
Brinkman Index, mean (SD)	820.4 (721)	732 (436)	0.689
Charlson Comorbidity Index, mean (SD)	1.37 (1.5)	1.66 (1.3)	0.316
Barthel Index, mean (SD)	98.5 (4.2)	97.4 (3.9)	0.205
MMSE, mean (SD)	27.9 (2.5)	25.4 (5.0)	<0.001
Stature (cm), mean (SD)	156.7 (9.4)	154.3 (12.1)	0.225
Body mass (kg), mean (SD)	58.6 (10.9)	51.8 (9.4)	0.001
Grip strength (kg), mean (SD)	26.3 (7.1)	20.8 (7.6)	<0.001
Gait speed (m/s), mean (SD)	1.2 (0.3)	0.97 (0.3)	<0.001
SMI (kg/m^2^), mean (SD)	6.7 (1.0)	6 (1)	<0.001
Pulmonary function test			
%VC (%), mean (SD)	95 (17.3)	85.5 (16.3)	0.007
FEV_1_/FVC (%), mean (SD)	78.7 (7.8)	80.6 (10.6)	0.273
Peak expiratory flow rate (L/min), mean (SD)	4.9 (1.6)	4.8 (2)	0.211

MMSE: Mini-Mental State Examination; SMI: skeletal muscle mass index; %VC: percent vital capacity; FEV_1_/FVC: forced expiratory volume in 1 s/forced vital capacity ratio; SD: standard deviation.

**Table 2:** Comparison of Diaphragm thickness, Thickening fraction, and Diaphragm excursion between non-sarcopenia and sarcopenia groups.

Parameter	Total	Non-Sarcopenia	Sarcopenia	Difference (95% CI)
Diaphragm thickness at FRC (mm), *n* = 136	2.04 (0.56)	2.03 (0.56)	2.05 (0.6)	0.02 (−0.2, 0.24)
Thickening fraction (%), *n* = 136	89.3 (54.1)	87.2 (50)	95.4 (65.0)	8.2 (−12.8, 29.2)
Diaphragm excursion (mm), *n* = 128	44 (14.2)	45.3 (14.7)	40.4 (12.3)	−4.9 (−10.5, 0.69)

Data are presented as the mean (SD). FRC: functional residual capacity; SD: standard deviation. CI: confidence interval. Sarcopenia includes participants who met the diagnostic criteria for sarcopenia of the AWGS 2019. Non-sarcopenia includes participants who did not meet the diagnostic criteria of the AWGS 2019. Difference was calculated as the mean of the sarcopenia group minus the non-sarcopenia group.

**Table 3:** Results of the simple and multiple regression analyses for sarcopenia and each right hemidiaphragm measurement.

(a). Simple regression analysis for right hemidiaphragm thickness, thickening fraction, and excursion						
Independent Variables Parameter	Diaphragm Thickness at FRC (mm), *n* = 136 Coefficient [95% CI] *p* Value		Thickening Fraction (%), *n* = 136 Coefficient [95% CI] *p* Value		Diaphragm Excursion (mm), *n* = 128 Coefficient [95% CI] *p* Value	
Sarcopenia (reference: non-sarcopenia)	0.02 [−0.2, 0.24]	0.85	8.2 [−12.8, 29.2]	0.44	−4.9 [−10.5, 0.7]	0.09
(b). Multiple regression analysis for each right hemidiaphragm measurement with adjustment for independent variables.						
Independent Variables Parameter	Diaphragm Thickness at FRC (mm), *n* = 136 Coefficient [95% CI] *p* Value		Thickening Fraction (%), *n* = 136 Coefficient [95% CI] *p* Value		Diaphragm Excursion (mm), *n* = 128 Coefficient [95% CI] *p* Value	
Sarcopenia (reference: non-sarcopenia)	0.004 [−0.23, 0.24]	0.98	12.1 [−10.2, 34.4]	0.28	−6.3 [−11.7, −0.85]	0.024 *
Age	0.007 [−0.008, 0.23]	0.35	−0.25 [−1.7, 1.2]	0.73	0.5 [0.16, 0.9]	0.005 *
Female sex (reference: male)	0.02 [−0.26, 0.3]	0.89	2.5 [−24, 29]	0.85	−1.2 [−7.7, 5.3]	0.7
Stature	0.009 [−0.005, 0.02]	0.224	1.1 [−0.23, 2.5]	0.1	0.56 [0.23, 0.9]	0.001 *

CI: confidence interval; FRC: functional residual capacity. Sarcopenia includes participants who met the diagnostic criteria for sarcopenia of the AWGS 2019. *: *p* < 0.05.

**Table 4:** Comparison of Diaphragm thickness, Thickening fraction, and Diaphragm excursion between Normal SMI and Low SMI groups.

Parameter	Total	Normal SMI	Low SMI	Difference (95% CI)
Diaphragm thickness at FRC (mm), *n* = 136	2.04 (0.56)	2.13 (0.6)	1.91 (0.54)	−0.22 (−0.41, −0.29)
Thickening fraction (%), *n* = 136	89.3 (54.1)	85 (49)	95 (60)	9.8 (−8.8, 28.4)
Diaphragm excursion (mm), *n* = 128	44.0 (14.2)	48.2 (14.7)	38.9 (11.8)	−9.2 (−14.0, −4.49)

Data are presented as the mean (SD). FRC: functional residual capacity; SD: standard deviation. CI: confidence interval. Low SMI includes participants who met the SMI cutoff defined in the diagnostic criteria for sarcopenia of the AWGS 2019. Normal SMI includes participants who did not meet the SMI cutoff defined in the diagnostic criteria for sarcopenia of the AWGS 2019. Difference was calculated as the mean of the sarcopenia group minus the non-sarcopenia group.

**Table 5:** Results of the simple and multiple regression analyses for skeletal muscle mass and each right hemidiaphragm measurement.

(a). Simple regression analysis for right hemidiaphragm thickness, thickening fraction, and excursion						
Independent Variables Parameter	Diaphragm Thickness at FRC (mm), *n* = 136 Coefficient [95% CI] *p* Value		Thickening Fraction (%), *n* = 136 Coefficient [95% CI] *p* Value		Diaphragm Excursion (mm), *n* = 128 Coefficient [95% CI] *p* Value	
Low SMI (reference: normal SMI)	−0.22 [−0.41, −0.29]	0.024 *	9.8 [−8.8, 28.4]	0.299	−9.2 [−14.0, −4.4]	<0.001 *
(b). Multiple regression analysis for each right hemidiaphragm measurement with adjustment for independent variables						
Independent Variables Parameter	Diaphragm Thickness at FRC (mm), *n* = 136 Coefficient [95% CI] *p* Value		Thickening Fraction (%), *n* = 136 Coefficient [95% CI] *p* Value		Diaphragm Excursion (mm), *n* = 128 Coefficient [95% CI] *p* Value	
Low SMI (reference: normal SMI)	−0.22 [−0.42, −0.017]	0.034 *	17.2 [−2.2, 36.5]	0.082	−8 [−12.7, −3.4]	0.001 *
Age	0.01 [−0.005, 0.24]	0.206	−0.2 [−1.6, 1.2]	0.789	0.5 [0.13, 0.82]	0.007 *
Female sex (reference: male)	−0.01 [−0.28, 0.26]	0.936	2.9 [−23.2, 29]	0.826	−1.5 [−7.8, 4.8]	0.639
Stature	0.005 [−0.09, 0.02]	0.473	1.3 [−0.05, 2.7]	0.058	0.5 [0.14, 0.81]	0.006 *

CI: confidence interval; FRC: functional residual capacity. Low SMI includes participants who met the SMI cutoff defined in the diagnostic criteria for sarcopenia of the AWGS 2019. *: *p* < 0.05.
